# Evaluation of ITGA3 as a Biomarker of Progression and Recurrence in Papillary Thyroid Carcinoma

**DOI:** 10.3389/fonc.2021.614955

**Published:** 2022-01-31

**Authors:** Guoliang Zhang, Bing Li, Yuanmei Lin

**Affiliations:** ^1^ Department of Thyroid Surgery, Affiliated Hospital of Putian University, Putian, China; ^2^ Department of Medical Oncology, Affiliated Hospital of Putian University, Putian, China

**Keywords:** papillary thyroid carcinoma (PTC), thyroid cancer, ITGA3, integrins, recurrence, prognosis, biomarker

## Abstract

**Objective:**

To investigate the expression of ITGA3 and its association with clinical outcomes in papillary thyroid carcinoma (PTC).

**Methods:**

The expression level, association with clinicopathologic characteristics, co-expressed genes, signaling pathways of ITGA3 in thyroid cancer were comprehensively analyzed using bioinformatics analysis through multiple public gene databases. PTC specimens and cell lines were used to verify the results of bioinformatics analysis.

**Results:**

Data mining based on the Oncomine database revealed that ITGA3 expression in classical PTC and tall cell variant PTC was much higher than that in normal thyroid tissue except the follicular variant PTC. Analysis based on The Cancer Genome Atlas (TCGA) database showed that the expression of ITGA3 varies greatly in pathological stages, pathological types, tumor invasion stages, and lymph node metastasis stages of thyroid carcinoma. High expression level of ITGA3 was correlated with tumor regional invasion and lymph node metastasis. Multivariate analysis using logistic regression model showed that high expression of ITGA3 was a risk factor that associated with PTC recurrence and lymph node metastasis. Survival analysis showed that patients with high expression of ITGA3 in PTC had a poorer relapse-free survival (RFS) than patients with low expression of ITGA3 (P < 0.05). Immunohistochemistry experiments showed that the expression of ITGA3 in recurrent thyroid cancer tissues was stronger than that in no-recurrent thyroid cancer tissues (P < 0.05). Knockdown of ITGA3 by sh-RNA in PTC cell lines suppresses cell viability and invasive and migrating capacity.

**Conclusion:**

ITGA3 is overexpressed in PTC, especially in those with higher tumor invasion grades and lymph node metastasis, and was associated with recurrence and poor RFS of PTC. High expression of ITGA3 may have the potential role of predicting PTC recurrence and lymph node metastasis.

## Introduction

Thyroid cancer is the most prevalent malignancy in the endocrine system (1). The most common subtype is papillary thyroid carcinoma (PTC), accounting for more than 80% of total thyroid malignant tumors (2). PTC is the least malignant of the four pathological types of thyroid cancer, and patients usually get a good long-term survival. However, PTC has a tendency to metastasize to the lymph nodes. It is reported that 30.0%–80.0% of patients have cervical lymph node metastasis at diagnosis (3, 4). Even for papillary thyroid microcarcinoma (PTMC), there were still 38.7% of the patients who have lymph node metastasis in the central area, and 16.7% of the patients have lateral lymph node metastasis (5). Whereas lymph node metastasis is closely connected with recurrence after surgical treatment (6, 7). Lee et al. (8) reported that the postoperative recurrence rate of papillary thyroid carcinoma was 1.4%. A retrospective study by Llamas-Olier et al. (9) found that the recurrence rate of T2-3N1 patients after surgery plus iodine-131 treatment still reached 7.4%. For patients who relapse after treatment with iodine-131, two-thirds will develop radioactive iodine-refractory thyroid cancer, and the overall prognosis is poor (10). At present, there is a lack of reliable indicators for predicting the prognosis of thyroid cancer in the clinic. Finding a predictive biomarker that associated with invasion and recurrence of PTC is of great significance, which may have a potential role in guiding clinical treatment.

Integrins (ITGs) are a class of membrane proteins with cell surface adhesion functions. Their main role is to mediate cell adhesion and promote tumor cell migration, proliferation, and survival (11). ITGA3 is an important member of the ITG family. Studies have shown that ITGA3 is highly expressed in multiple cancers and is related to tumor progression and poor prognosis (12–14).

In this study, Oncomine database was used to examine the expressions of ITGA3 in PTC and normal thyroid tissue. Analysis based on The Cancer Genome Atlas (TCGA) database was made to explore the correlation between PTC and its clinicopathologic characteristics. Signaling pathways related to ITGA3 were also analyzed that might be relevant in PTC progression and prognosis. PTC specimens and cell lines were used to verify the results of bioinformatics analysis.

## Materials and Methods

### Analysis of ITGA3 Expression in Multicancers

The mRNA expression levels of ITGA3 in multicancers and their normal tissue counterparts were analyzed using the Oncomine database (https://www.oncomine.org/) (15) and GEPIA database (http://gepia.cancer-pku.cn) (16, 17). ITGA3 expression patterns were obtained by queries with default settings in all analyses with these databases.

### Analysis of ITGA3 Expression in PTC and Its Normal Tissue

Data mining based on Oncomine database (http://www.oncomine.org/) was used to examine the expression difference of ITGA3 gene in papillary thyroid carcinoma. Oncomine is currently the world’s largest oncogene chip database and integrated data mining platform for researchers to find genes that are differentially expressed in a certain type of cancer. In this study, the screening conditions we set were as follows: “Cancer Type: papillary thyroid cancer”; “Gene: ITGA3”; “Analysis Type: Cancer vs Normal Analysis.” R software and ggplot2 package were used for charting.

### ITGA3 Expression in The Cancer Genome Atlas Dataset With Different Clinicopathologic Characteristics

ITGA3 mRNA expression in thyroid cancer and its association with each clinicopathologic characteristic were analyzed in TCGA thyroid cancer cohort (18). ITGA3 mRNA expression in thyroid cancer samples and characteristics of cancer stage, lymph node stage, histological subtype were separately analyzed. “R” software and “ggplot2”and “ggpubr” package were used for statistical analysis and charting.

### Diagnostic Value of ITGA3 in PTC Progression

The diagnostic value of ITGA3 in PTC progression was evaluated using receiver operating characteristic (ROC) analysis. The sensitivity, specificity, area under the curve (AUC), and the best cutoff value were calculated in the ROC analysis to evaluate the prediction value of ITGA3 for lymph node metastasis, local invasion, and recurrence. The best cutoff value was determined based on Youden index. “R” software and “pROC” package were employed to assist in the analysis.

### Prognostic Value Assessment for ITGA3 in PTC

The prognostic value of ITGA3 in PTC was determined using survival analysis. The relapse-free survival (RFS) rate was analyzed using Kaplan–Meier model. ROC analysis was used to determine the best cutoff of ITGA3 expression for predicting recurrence of PTC, on which the patients were divided into low expression and high expression groups. Then, univariate and multivariate analysis of RFS was employed using Cox regression model with the purpose of probing the independent risk factors that may influence recurrence for patients with PTC. “R” software and “survival,” “survminer,” “ggplot2”packages were used for statistical analysis and charting.

### Analysis of Genes Coexpressed With ITGA3 in PTC

To further investigate the potential regulative mechanism of ITGA3 in PTC, we explored genes that were co-expressed with ITGA3 in thyroid cancer using the Gene Expression Profiling Interactive Analysis (GEPIA) database (http://gepia.cancer-pku.cn), in which the tumor samples were from TCGA database and the normal samples were from both TCGA and GTEx projects (18). The correlation between ITGA3 and its top 20 positively co-altered genes was illustrated with heat map using UCSC Xena web (19). Scatter plot was constructed for the most significant gene using both GEPIA and UCSC Xena web.

### Enrichment Pathway Analysis Based on ITGA3 Expression

Gene Set Enrichment Analysis (GSEA) is a powerful analytical method for functional interpretation of gene expression data (20, 21). In this analysis, Broad Institute GSEA software (version 4.1.0) was applied to identify signaling pathways related to ITGA3. TCGA data of thyroid cancer were used for this analysis. Patients were divided into low and high expression groups according to ITGA3 expression level, and 1,000 permutations were set as a normalized enrichment score (NES). The screening thresholds considered significant were set as P-value <0.05, false discovery rate (FDR) <0.1.

### Immunohistochemistry Assay

A total of 58 thyroid cancer samples that were embedded in paraffin were collected for immunohistochemistry assay, including 30 cases of no-recurrent PTC and 28 cases of recurrent PTC of our hospital in the recent 10 years. These paraffin-embedded tissues are processed into tissue microarray. The tissue sections were incubated with ITGA3 mouse polyclonal antibody (1:1,000; Kanglang Biotech, Shanghai) overnight. After washing with phosphate buffered saline (PBS) thrice, those sections were incubated with secondary antibodies conjugated to horseradish peroxidase-labeled polymers. Finally, those sections were counterstained with hematoxylin. ITGA3 expression level was determined by immunoreactive score (IRS), which was obtained by multiplying the score of staining intensity with the score of positive area. The staining intensity was scored as follows: negative (score 0), weak (score 1), moderate (score 2), and strong (score 3). The score of positive cells was defined as follows: negative (score 0), 0%–25% (score 1), 26%–50% (score 2), 51%–75% (score 3), and >76% (score 4). IRS scores of 0–4 were defined as low, 5–8 were defined as moderate, and >8 were defined as high expression.

### PTC Cell Line Culture and sh-RNA Interference

The human PTC cell line BCPAP was purchased from OUTDO BIOTECH. All cells were cultured in Dulbecco's Modified Eagle Medium (DMEM) medium containing 10% fetal bovine serum (FBS) and 1% penicillin-streptomycin. Four short hairpin RNA plasmids targeting ITGA3 (shRNA-ITGA3) and corresponding negative control (shRNA-NC) were transferred into cells. The targeted sequences were listed as follows: sh-ITGA3-2625: 5′-GGTAAATCACCGGCTACAAAG-3′, sh-ITGA3-3137: 5′-GCACCTTCATCGAGGATTACA-3′, sh-ITGA3-2276: 5′-GGAAATTGCTCCTGAGCATCA-3′, sh-ITGA3-884: 5′-GCAATAGCAACACAGACTACC-3′. After the transfection, RT-PCR and Western blot assay were performed to examine the infection efficiency. Primer sequences were designed as follows: F: 5′-CTTCCACGGCTTCTTCTCCA-3′, R: 5′-GGGCATCCGCAAAGGTAAAGA-3′. 5-bromo-4-chloro-3-indolyl phosphate/nitroblue tetrazolium chloride (BCIP/NBT) alkaline phosphatase was used to detect protein transmembrane and expression.

### Cell Counting Kit-8 Proliferation Assay

In the Cell Counting Kit-8 (CCK8) assay, BCPAP was seeded into 96-well plates with 5,000 cells per well. Cell proliferation was detected after 24 and 72 h, respectively. The absorbance of each well was detected by a microplate reader at a wavelength of 450 nm.

### Cell Invasion and Migration Assay

Wound scratch assay and transwell invasion assay were conducted to examine the alteration of cell viability, cell migratory capacity, and cell invasion capacity. In the wound scratch assay, cells were seeded into 6-well plates with 1 × 10^5^ cells per well in each group. After culturing to 100% confluence, a 1-mm-width scratch in each group was made with a plastic scriber. Wells were washed with PBS to remove the floating cell. Scratch repair was observed under an inverted microscope after culturing for 12, 24, and 48 h. In the cell invasion assay, Transwell Chambers precoated with Matrigel were used to examine cell invasion capacity. A total of 200 µl cell suspension with a density of 1 × 10^6^ cells/ml in each group were added to the upper chamber, while 500 µl DMEM with 10% FBS were added to the lower chamber. After 24 h incubation at 37°C with 5% CO_2_, cells that did not invade through the pores were wiped out using a micropipette tip. The filters were stained using 0.1% crystal violet, and the invasive cell number was determined from five randomly selected fields under an inverted microscope.

### Statistical Method

The counting data were analyzed by chi-square test. The measurement data were analyzed by parameter test. Student’s t test was used for the comparisons between two groups. One-way ANOVA test was used for comparisons of more than or equal to three groups. Student’s t test was used for pairwise comparison in ANOVA. Binary logistic regression model was used for multivariate analysis to determine independent risk factors that affect tumor recurrence. Kaplan–Meier model was used for survival analysis. P < 0.05 was considered statistically significant.

## Results

### ITGA3 mRNA Is Highly Expressed in Multiple Cancers

**Figure 1:**
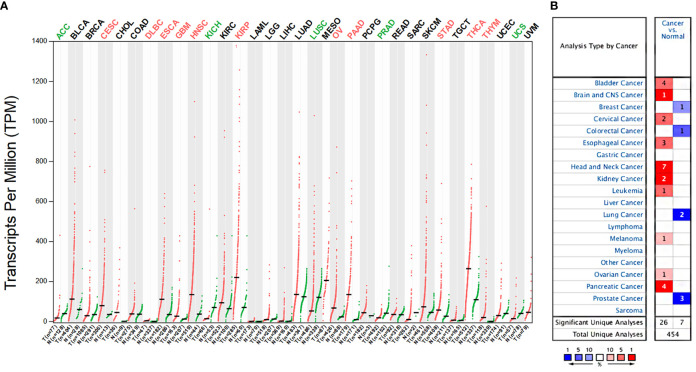
ITGA3 mRNA expression levels in different types of human cancers. **(A)** The expression of ITGA3 in pan-cancer in The Cancer Genome Atlas (TCGA) database was obtained from GEPIA database. **(B)** The expression of ITGA3 in different cancers compared with normal tissues in the Oncomine database.

We examined the expression differences of ITGA3 between cancers and their normal tissues using GEPIA and Oncomine databases to explore the expression pattern in multicancers. Analysis of the expression of ITGA3 in pan-cancer and its normal tissues based on TCGA datasets using GEPIA web-based tool revealed that 11 cancers displayed significantly higher ITGA3 expression and 5 cancers displayed lower ITGA3 expression compared to their normal tissues (**Figure 1A**). In the Oncomine database, ITGA3 gene was highly expressed in head and neck, kidney, bladder, pancreatic, and esophageal cancers and so on, with 26 datasets involved in the group. Only 4 types of cancers revealed the low expression of ITGA3 including breast, lung, colorectal, and prostate cancers, with 7 datasets involved in the group (**Figure 1B**).

### ITGA3 Is Highly Expressed in Papillary Thyroid Carcinoma Based on Oncomine Database

**Figure 2:**
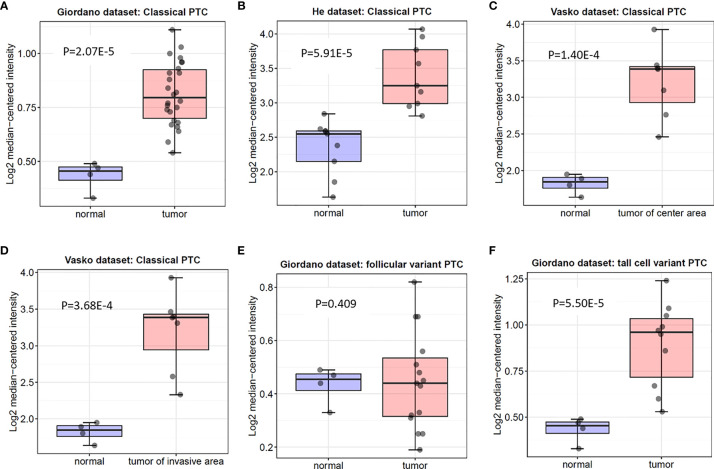
Box plot of ITGA3 expression in normal thyroid gland and papillary thyroid cancer using the Oncomine database. P−values were calculated by Student’s t test. **(A–D)** The expression of ITGA3 in normal thyroid gland and classical papillary thyroid cancer. **(E)** The expression of ITGA3 in normal thyroid gland and follicular variant papillary thyroid cancer. **(F)** The expression of ITGA3 in normal thyroid gland and tall cell variant papillary thyroid cancer.

In the Oncomine database, there are 3 distinct datasets involving the expression of ITGA3 in papillary thyroid carcinoma and normal thyroid tissue, which contains 6 subsets and 74 tumor samples. The expression level of ITGA3 is much higher in PTC tissues than that in normal thyroid tissues in 5 of the subsets, except the follicular variant papillary carcinoma subset (**Figure 2** and **Table 1**).

**Table 1:** ITGA3 expression in PTC in Oncomine database.

Pathology	Gene	Sample number	Fold change	t test	P-value	Reference
Papillary carcinoma of center area	ITGA3	7	2.74	7.11	1.40E-4	PMID 17296934
Papillary carcinoma of invasive area	ITGA3	7	2.75	6.29	3.68E-4	PMID 17296934
Papillary carcinoma	ITGA3	9	2.053	5.083	5.91E-5	PMID 16365291
Papillary carcinoma	ITGA3	26	1.300	8.257	2.07E-5	PMID 16609007
Tall cell variant Papillary carcinoma	ITGA3	10	1.376	5.674	5.50E-5	PMID 16609007
Follicular variant Papillary carcinoma	ITGA3	15	1.010	0.234	0.409	PMID 16609007

Statistical significance was determined by the Student’s t test. P < 0.05 was considered as the difference that was statistically significant.

### ITGA3 Gene Expression Is Correlated With Clinicopathologic Characteristics Including Tumor Stage, Pathological Subtypes, Tumor Invasion, and Lymph Node Stage

**Figure 3:**
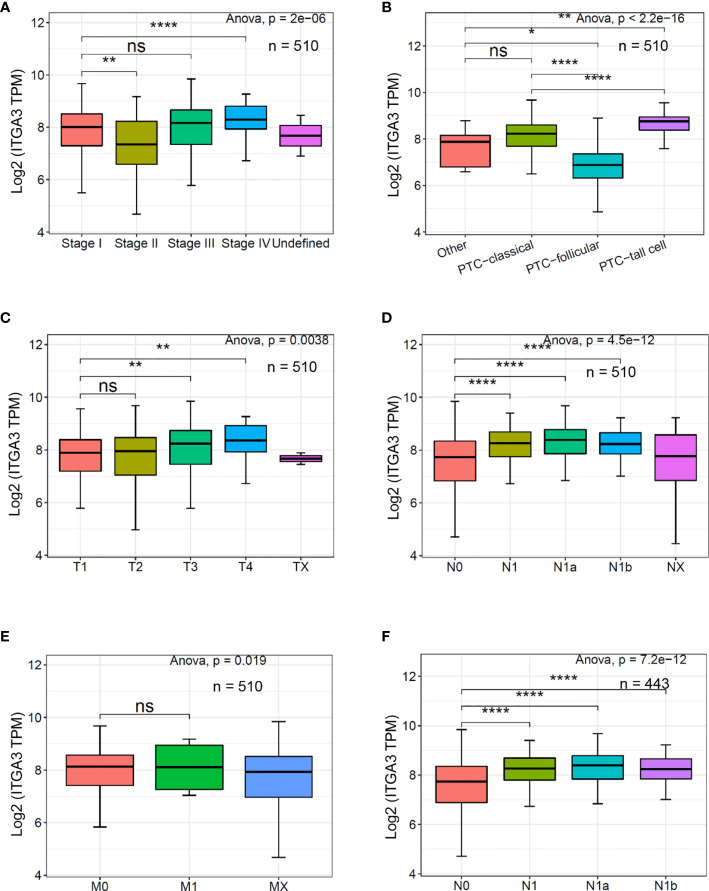
The expression of ITGA3 in thyroid cancer with different clinicopathologic characters based on The Cancer Genome Atlas (TCGA) database. **(A–E)** The expression of ITGA3 in different pathologic stages, histological types, pathologic T stages, pathologic N stages, and pathologic M stages. **(F)** The expression of ITGA3 in different pathologic N stages in papillary thyroid cancer excluding the cases of non-papillary thyroid carcinoma and undefined histological types. Statistical significance was determined by one-way ANOVA test. P < 0.05 was considered as the difference that was statistically significant (*P < 0.05, **P < 0.01,****P < 0.0001). ns, no significance.

ITGA3 gene expression was significantly correlated with clinicopathologic characteristics in TCGA thyroid cancer dataset. Firstly, we explored the expression of ITGA3 in different tumor stages. It turned out that ITGA3 in stage IVA was significantly greater than that in stage I (P < 0.01). Subsequently, we grouped these cases as classical PTC, follicular variant PTC, tall cell variant PTC, and other histologic type according to the histological subtypes. The comparison suggested that tall cell variant PTC expressed the highest level of ITGA3, followed by classical PTC, and the lowest level was in follicular subtype (P < 0.01). Considering that the stage of thyroid cancer was greatly influenced by age, tumor stage cannot accurately reflect tumor invasion and lymph node metastasis. Therefore, we grouped these cases according to T stage and lymph node stage. The analysis showed that the expression levels of ITGA3 in T3 and T4 stages are significantly higher than those in T1 stage. ITGA3 expression in cases with lymph node metastasis was significantly higher than that in cases without lymph node metastasis (P < 0.01). There is no difference between M0 and M1 stages in the expression of ITGA3. Finally, we only enrolled the pathological type of PTC from the dataset, then the comparison based on lymph node stage was conducted. The results showed that the expression level of ITGA3 in the group with lymph node metastasis was significantly higher than that without lymph node metastasis group in PTC (P < 0.01) (**Figure 3**).

### High Expression of ITGA3 Is Associated With the Recurrence and Lymph Node Metastasis of Papillary Thyroid Carcinoma

Univariate analysis for PTC recurrence suggested that high expression of ITGA3, histologic type, pathologic T, and pathologic N were significantly correlated with the recurrence of PTC (**Table 2**). Hence, the four variables were included in the following multivariate logistic regression analysis. The results indicated that ITGA3 [P = 0.020, odds ratio (OR) = 1.003] and pathologic T (P = 0.042, OR = 3.815) were the risk factors that associated with the recurrence of PTC (**Table 3**).

**Table 2:** Univariate analysis for PTC recurrence in the cohort of TCGA data.

Risk factors	Recurrence		t/χ^2^	P-value
	Yes (n = 30)	No (n = 415)		
ITGA3 (log2TPM), mean (SD)	8.26 (0.99)	7.81 (1.00)	-2.40	0.022
Gender, n (%)			5.42e-05	0.83
Male	8 (26.70)	103 (24.80)		
Female	22 (73.30)	312 (75.20)		
Age, mean (SD)	45.77 (16.12)	46.48 (15.52)	0.23	0.817
Pathologic stage, n (%)			2.82	0.495
I	15 (50.00)	250 (60.24)		
II	3 (10.00)	46 (11.08)		
III	10 (33.33)	85 (20.48)		
IV	2 (6.67)	33 (7.95)		
Undefined	0 (0)	1 (0.2)		
Histologic type, n (%)			7.49	0.033
Classical PTC	20 (66.67)	296 (71.33)		
Tall cell variant PTC	4 (13.33)	91 (21.93)		
Follicular variant PTC	6 (20.00)	28 (6.75)		
Pathologic T, n (%)			12.43	0.016
T1	3 (10.00)	136 (32.77)		
T2	10 (33.33)	145 (34.94)		
T3	17 (56.67)	121 (29.16)		
T4	0 (0)	11 (2.65)		
TX	0 (0)	2 (0.48)		
Pathologic N, n (%)			6.23	0.039
N0	8 (26.70)	201 (48.40)		
N1	19 (63.30)	169 (40.70)		
NX	3 (10.00)	45 (10.80)		
Pathologic M, n (%)			4.50	0.100
M0	14 (46.67)	241 (58.07)		
M1	1 (3.33)	2 (0.48)		
MX	15 (50.00)	172 (41.45)		

SD, standard deviation; TPM, transcripts per million; PTC, papillary thyroid carcinoma.

Statistical significance (P < 0.05).

**Table 3:** Binary logistic regression analysis with multiple factors for thyroid cancer recurrence.

Variables	B	Wald	P	OR	95% CI
ITGA3	0.003	5.458	0.0195	1.003	1.001–1.006
Pathologic N (N1)	0.898	3.405	0.0650	2.454	0.984–6.788
Pathologic T (T3)	1.339	4.129	0.0421	3.815	1.180–17.081
Histologic type (tall cell variant)	0.647	1.342	0.2467	1.911	0.598–5.504

CI, confidence interval; OR, odds ratio.

Statistical significance (P < 0.05).

We adopted the same methods to explore the predictive value of ITGA3 for PTC lymph node metastasis. The analysis suggested that high expression of ITGA3 (P = 0.0105, OR = 1.002) and pathologic T3 (P = 0.0002, OR = 2.974) and T4 (P = 0.0053, OR = 10.578) were the risk factors. Besides, follicular variant PTC (P = 0.0002, OR = 0.233) and age ≥55 (P = 0.0003, OR = 0.38) were the risk factors negatively associated with lymph node metastasis (**Table 4**).

**Table 4:** Binary logistic regression analysis with multiple factors for thyroid cancer lymph node metastasis.

Variables	B	Wald	P	OR	95% CI
ITGA3	0.002	6.543	0.0105	1.002	1.001–1.004
Age (≥55 years)	-0.967	13.09	0.0003	0.38	0.223–0.637
Gender	0.398	2.243	0.1342	1.489	0.886–2.519
Pathologic T (T3)	1.090	13.773	0.0002	2.974	1.682–5.331
Pathologic T (T4)	2.359	7.788	0.0053	10.578	2.352–75.547
Histologic type (follicular variant)	-1.459	1.342	0.0002	0.233	0.104–0.484

CI, confidence interval; OR, odds ratio.

Statistical significance (P < 0.05).

### The Diagnostic Performance of ITGA3 in PTC

**Figure 4:**
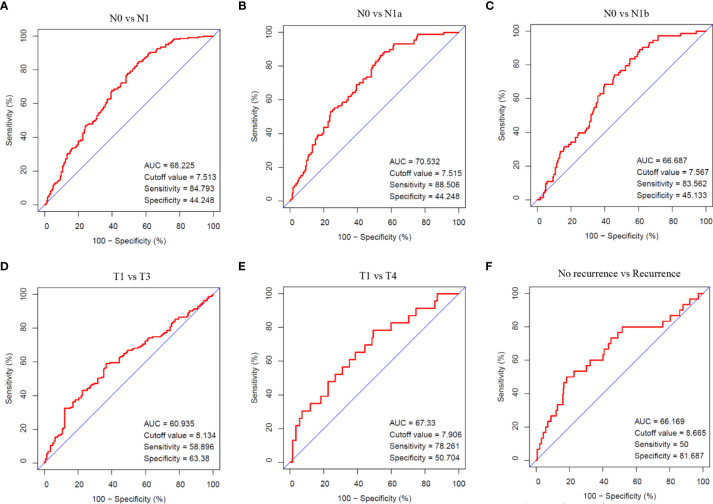
The diagnostic value of ITGA3 in PTC progression using ROC analysis. **(A)** Comparison between N0 and N1 stages. **(B)** Comparison between N0 and N1a stages. **(C)** Comparison between N0 and N1b stages. **(D)** Comparison between T1 and T3 stages. **(E)** Comparison between T1 and T4 stages. **(F)** Comparison between no recurrence and recurrence groups. AUC, Area under curve.

Cases with pathologic type of papillary thyroid carcinoma (including classical, tall cell variant, and follicular variant papillary thyroid carcinoma) were screened out from TCGA thyroid cancer cohort. Comparisons between N0 and N1, N0 and N1a, N0 and N1b, T1 and T3, T1 and T4, no recurrence and recurrence in PTC were conducted using ROC analysis. The indexes in these comparisons were AUC 68.225, cutoff value 7.513, sensitivity 84.793, and specificity 44.248 in N0 vs. N1; AUC 70.532, cutoff value 7.515, sensitivity 88.506, and specificity 44.248 in N0 vs. N1a; AUC 66.687, cutoff value 7.567, sensitivity 83.562, and specificity 45.133 in N0 vs. N1b; AUC 60.935, cutoff value 8.134, sensitivity 58.896, and specificity 63.38 in T1 vs. T3; AUC 67.33, cutoff value 7.906, sensitivity 78.261, and specificity 50.704 in T1 vs. T4; and AUC 66.169, cutoff value 8.665, sensitivity 50, and specificity 81.687 in no recurrence vs. recurrence, respectively (**Figure 4**). The analysis indicated that the expression of ITGA3 had a moderate diagnostic significance for lymph node metastasis in neck central area and low diagnostic significance for regional invasion and recurrence.

### The Prognostic Value of ITGA3 in PTC

**Figure 5:**
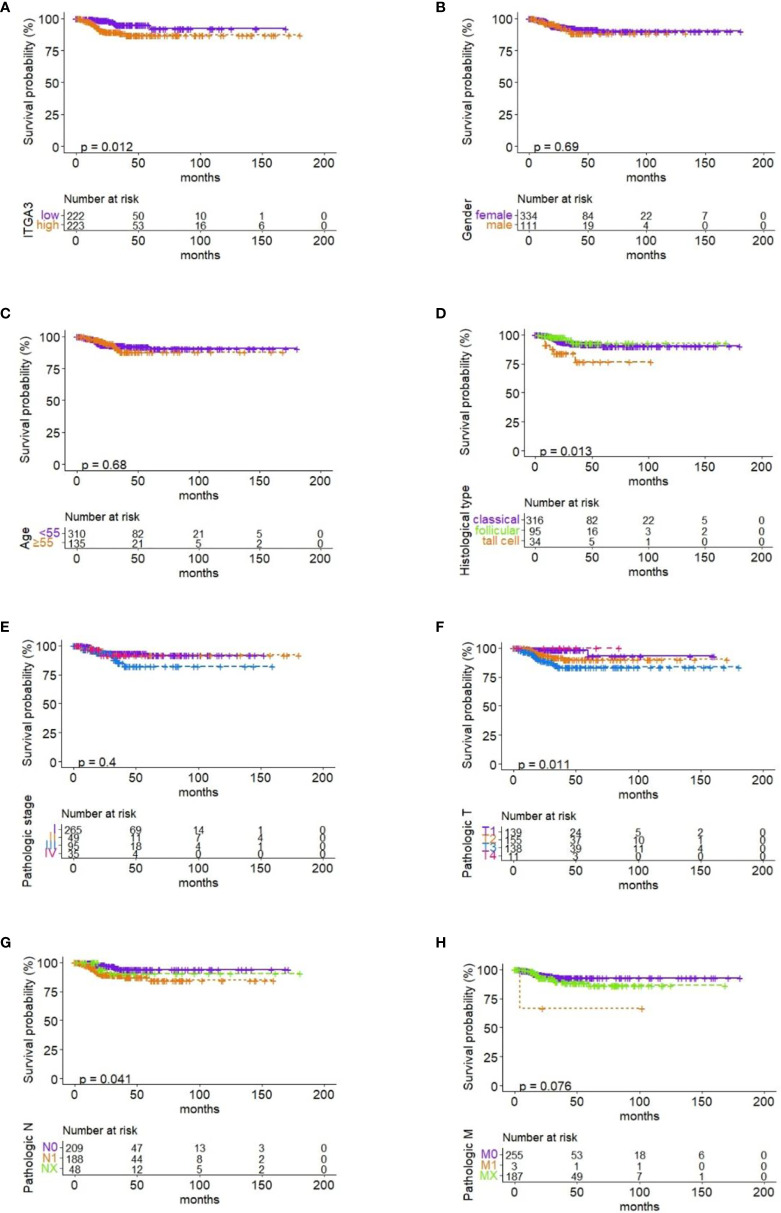
Correlation between RFS and ITGA3 expression and clinicopathologic characteristics. **(A)** Survival analysis of RFS by ITGA3 expression with the median level. **(B)** Survival analysis of RFS by gender. **(C)** Survival analysis of RFS by age. **(D)** Survival analysis of RFS by pathologic subtype. **(E)** Survival analysis of RFS by pathologic stage. **(F)** Survival analysis of RFS by pathologic T. **(G)** Survival analysis of RFS by pathologic N. **(H)** Survival analysis of RFS by pathologic M.

Firstly, the patients were divided into low expression and high expression groups on medium expression value of ITGA3. Then, survival analysis of RFS in PTC with ITGA3 expression was conducted using Kaplan–Meier model. The analysis showed that high expression of ITGA3 was connected with poor RFS (P = 0.012; **Figure 5A**).

To investigate the effect of clinicopathologic characteristics on recurrence, we conducted survival analysis of RFS based on different clinicopathologic characteristics. The results showed that histological type, pathologic T, and pathologic N were associated with RFS in PTC (P ≤ 0.05; **Figures 5D, F, G**). Gender, age, pathologic stage, and pathologic M have no significant association with RFS (**Figures 5B, C, E, H**).

### Co-Expressed Profile With ITGA3 in Thyroid Cancer

**Figure 6:**
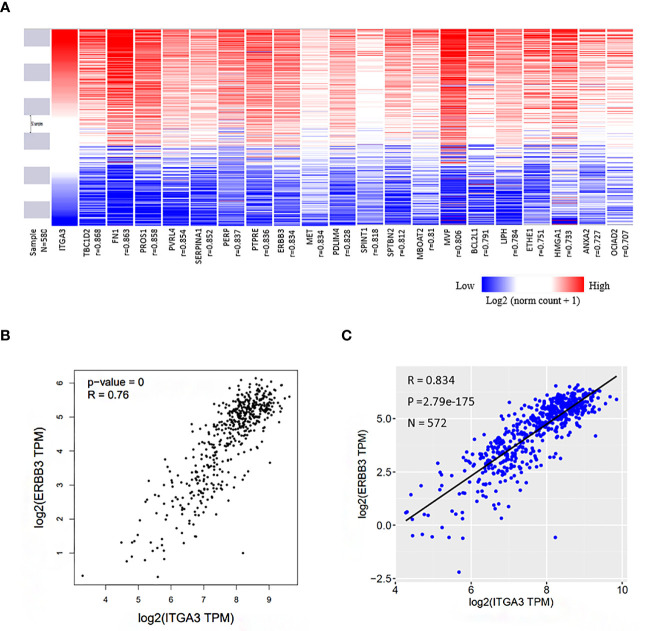
Co-expression genes of ITGA3 in thyroid cancer. **(A)** Heat map of the co-expression profile of ITGA3 in thyroid cancer plotted by the UCSC Xena web-based tools. The co-altered gene list was obtained from GEPIA database in which the dataset of thyroid cancer was also from TCGA. **(B)** Correlation between ITGA3 and ERBB3 in GEPIA database. **(C)** Correlation between ITGA3 and ERBB3 expression in thyroid carcinoma using the UCSC Xena web.

We identified a set of genes that were positively co-expressed with ITGA3 in thyroid cancer using GEPIA database, in which the tumor samples originated from TCGA database. Heat map of the co-expression profile of ITGA3 in thyroid cancer was plotted by the UCSC Xena web-based tools (**Figure 6A**). We found that Erb-B2 receptor tyrosine kinase 3 (ERBB3), also known as HER3, was highly correlated with ITGA3 among the profile. The correlation between ITGA3 and ERBB3 was confirmed by scatter plot using GEPIA database and UCSC Xena web (**Figures 6B, C**). Our findings suggested that the expression of ITGA3 and ERBB3 were closely correlated and may contribute to a signaling pathway in thyroid cancer.

### Enriched Signaling Pathways Related to ITGA3 High Expression in Thyroid Cancer

**Figure 7:**
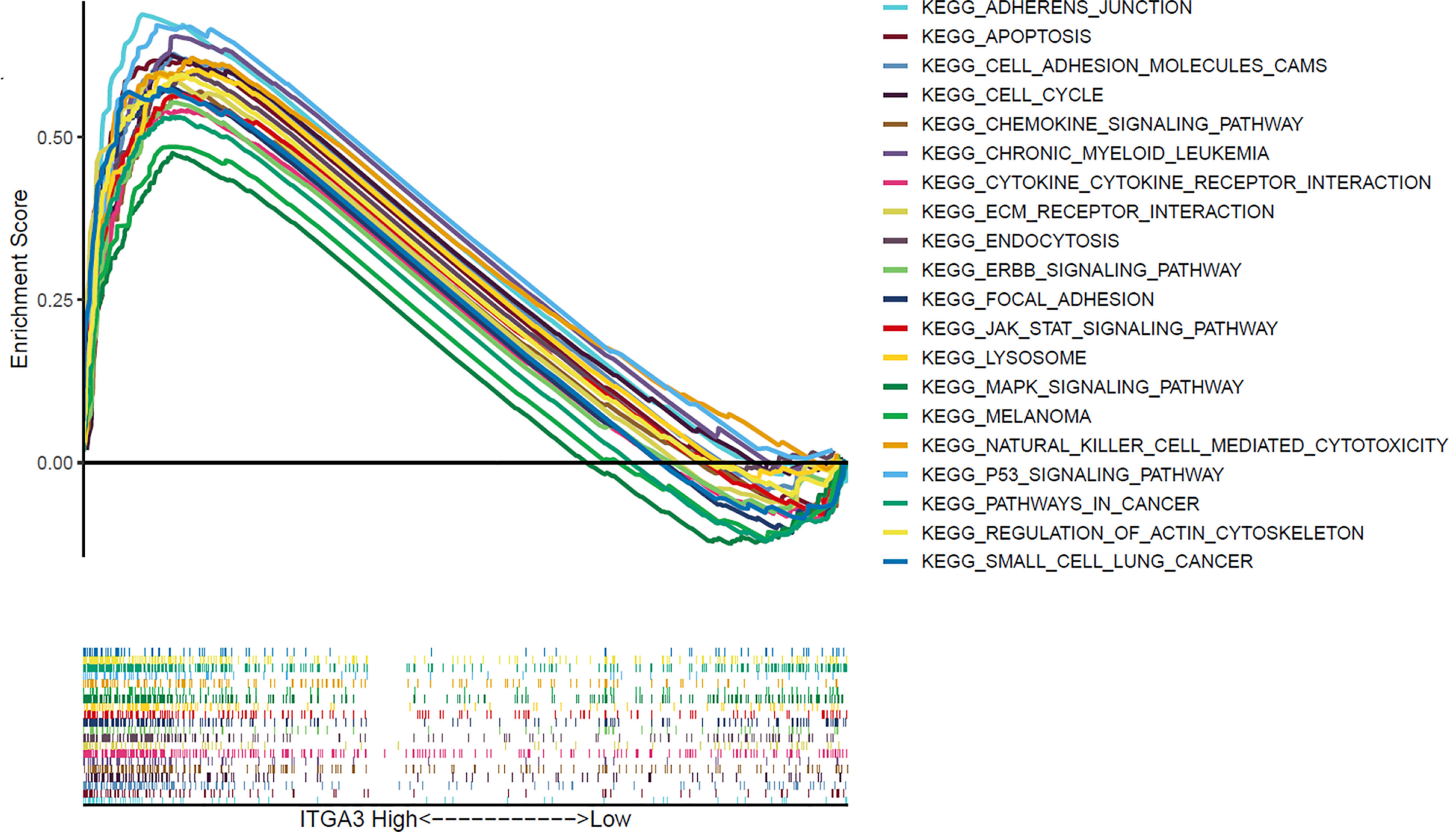
Gene set enrichment analysis of signaling pathway based on ITGA3 expression in TCGA cohort of thyroid cancer.

GSEA performed using TCGA data revealed that several cancer-related pathways were linked to ITGA3 high expression, including apoptosis, cell cycle, chronic myeloid leukemia, ERBB signaling pathway, mitogen-activated protein kinase (MAPK) signaling pathway, melanoma, P53 signaling pathway, pathways in cancer, and small cell lung cancer. Besides, extracellular matrix (ECM) receptor pathway and focal adhesion pathway, which was associated with cell survival and migration, were also enriched in the ITGA3 high expression group (**Figure 7**).

### Validation of the Expression of ITGA3 in PTC and Adjacent Normal Tissues With Immunohistochemistry

**Figure 8:**
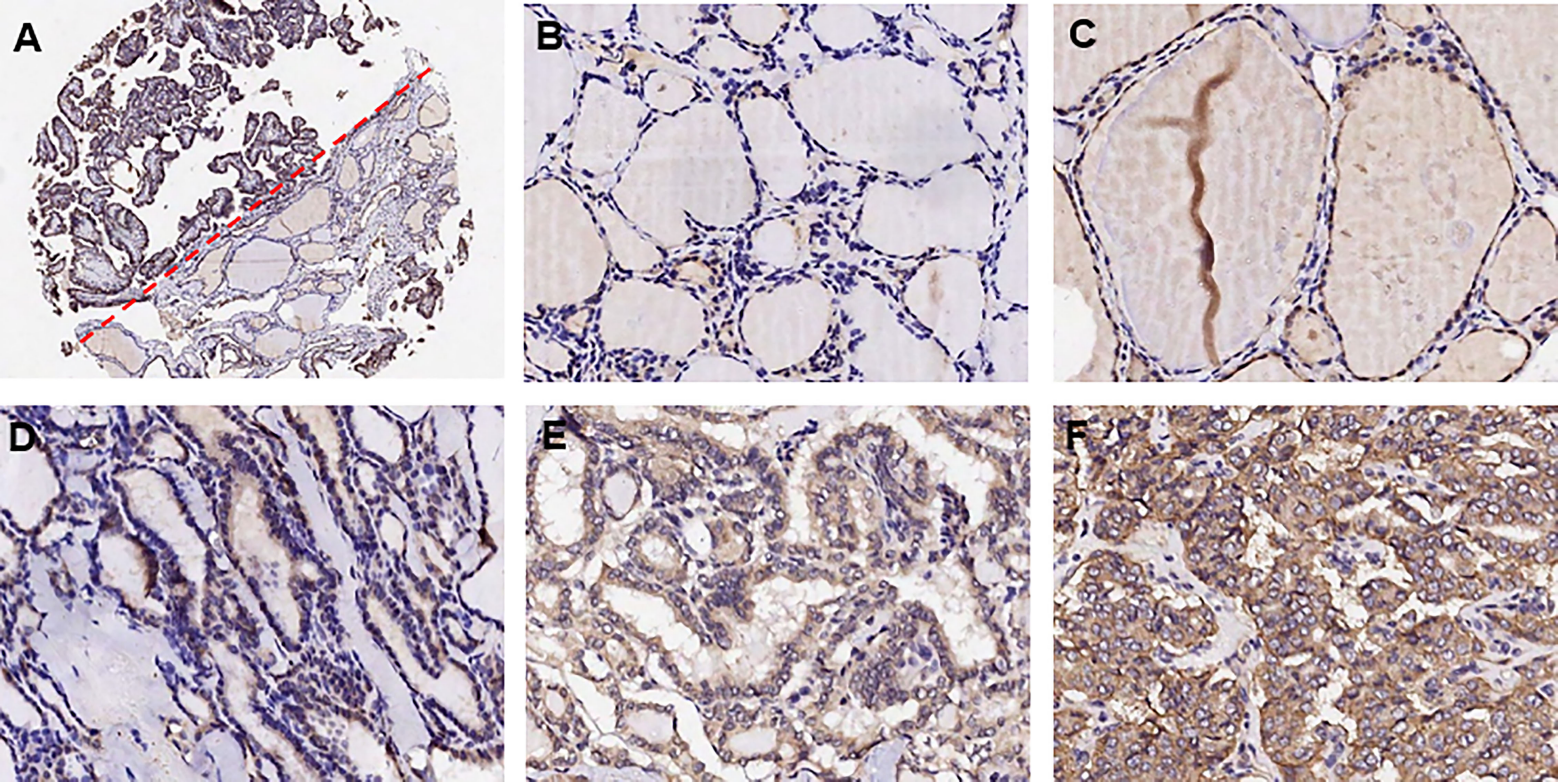
Detection of ITGα-3 in papillary thyroid cancer and adjacent normal gland. ITGα-3 is mainly located in the cytoplasm. **(A)** Contrast of staining intensity between normal gland and PTC. **(B)** Weak staining of ITGα-3 in normal gland. **(C)** Moderate staining of ITGα-3 in normal gland. **(D)** Weak staining of ITGα-3 in PTC. **(E)** Moderate staining of ITGα-3 in PTC. **(F)** Strong staining of ITGα-3 in PTC.

We adopted the immunohistochemistry method to detect the ITGα-3 (protein of ITGA3) expression in papillary thyroid cancer and adjacent normal gland. As shown in **Figure 8**, ITGα-3 is mainly located in the cytoplasm, and the expression of ITGα-3 in thyroid cancer tissues was significantly stronger than that in normal gland. Subgroup analysis showed that the expression of ITGα-3 in the group of recurrent PTC was much higher than that in the group of no-recurrent PTC (**Table 5**).

**Table 5:** Correlation between the clinicopathologic characteristics and expression of ITGA3.

Parameters	ITGα-3 expression			P
	Low	Moderate	High	
Age (years)				0.619
<55	3	19	6	
≥55	1	22	7	
Gender				0.74
Male	2	15	6	
Female	2	26	7	
Histological type				2.2e-16
Normal	57	1	0	
No-recurrence	4	23	3	0.011*
Recurrence	0	18	10	
Pathologic T				0.045
T1	2	8	0	
T2	1	10	1	
T3	1	13	4	
T4	0	10	8	
Pathologic N				0.134
N0	3	8	2	
N1a	1	17	4	
N1b	0	16	7	

Chi-square test, *comparison between no-recurrence and recurrence groups, statistical significance (P < 0.05).

### Validation of Transfection Efficiency With shRNA

**Figure 9:**
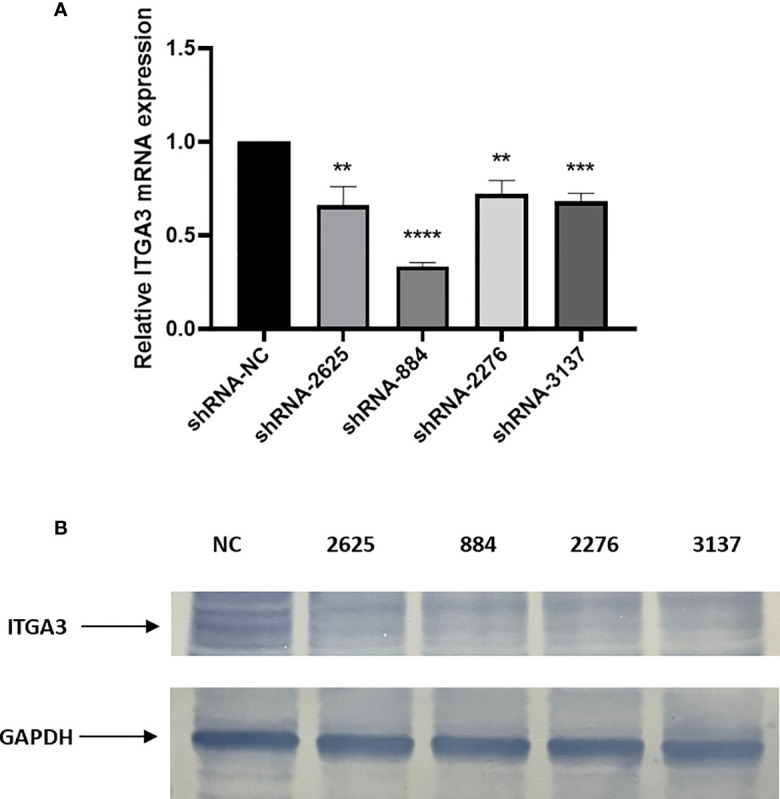
Knockdown of ITGA3 inhibits proliferation of PTC cells *in vitro*. **(A)** shRNA transfection efficiency analysis with RT-PCR. **(B)** Western blot test of the expression of ITGA3 protein after transfection (****P < 0.0001, ***P < 0.001, **P < 0.01, compared with negative control).

We adopted RT-PCR and Western blot assays to verify the transfection efficiency with shRNA. The results showed that sh-RNA-884 has the greatest transfection efficiency that inhibited ITGA3 expression mostly (**Figures 9A, B**). Therefore, sh-RNA-884 was selected for the following experiments.

### The Impact of ITGA3 Knockdown on PTC Cell Viability

**Figure 10:**
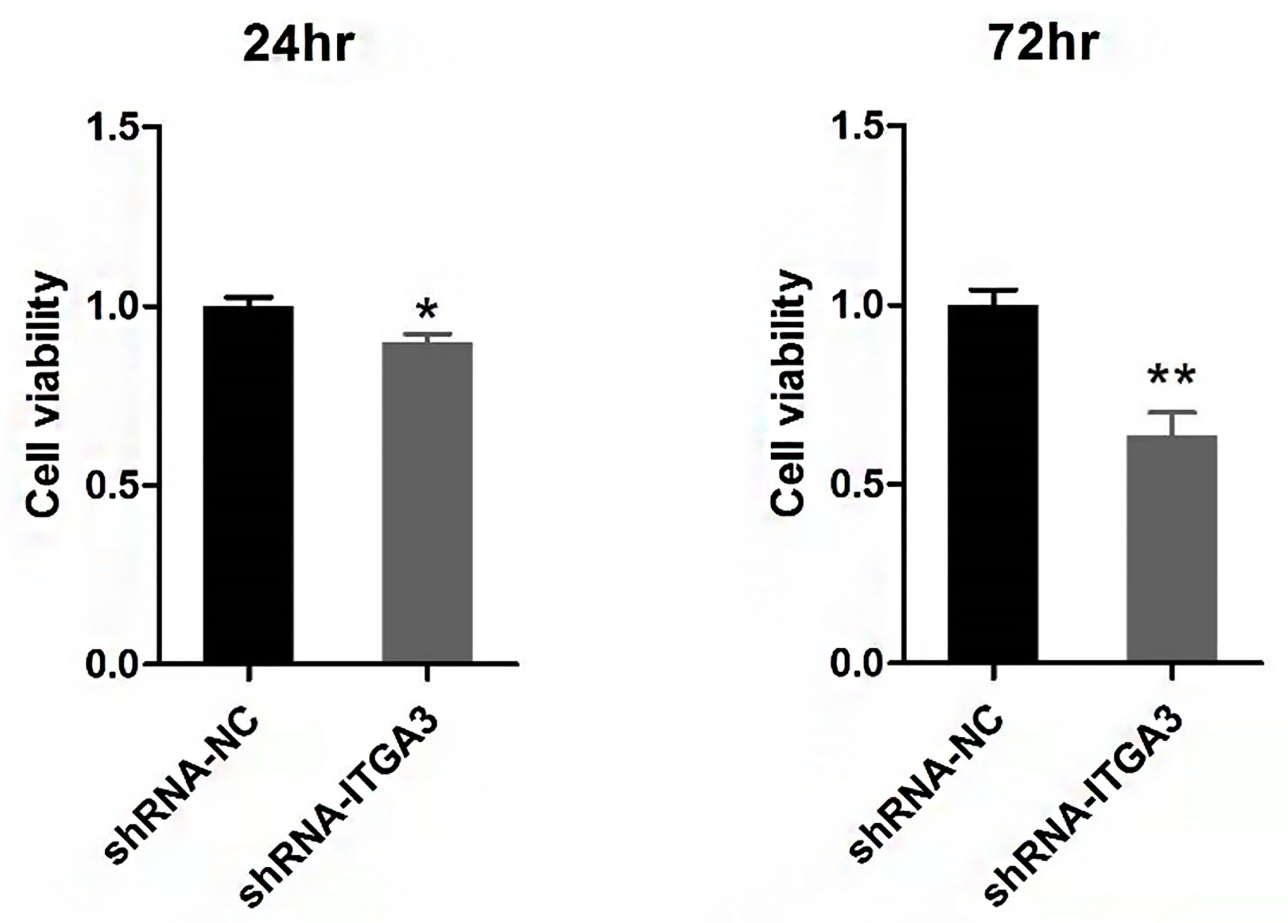
Viability analysis of BCPAP cells with CCK8 assay (**P < 0.01, *P < 0.05, compared with negative control).

We examined the effect of ITGA3 knockdown on the growth of PTC cells *in vitro* to evaluate the role of ITGA3 on PTC cell viability. Sh-RNA-884 plasmid targeting ITGA3 was transfected to BCPAP cells to decrease the expression of ITGA3. The corresponding sh-NC plasmid transfection was used as control. The results showed that knockdown of ITGA3 inhibits the proliferation of BCPAP cells (**Figure 10**).

### Knockdown of ITGA3 Inhibits Migration and Invasion of PTC Cells *In Vitro*

**Figure 11:**
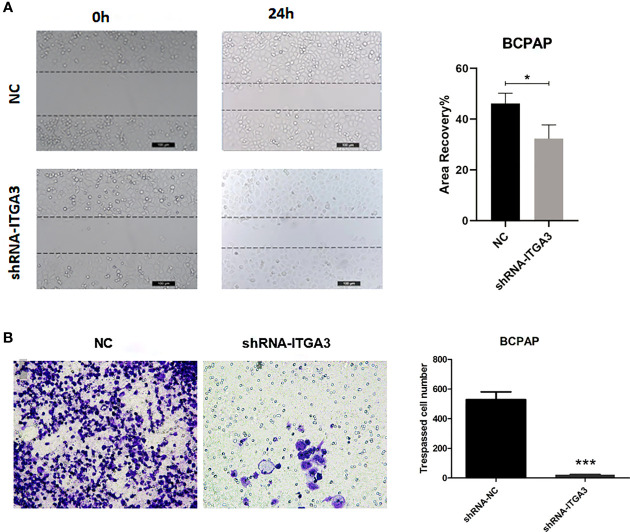
The knockdown of ITGA3 levels significantly inhibits the capacity of migration and invasion of renal cancer cells *in vitro*. **(A)** Wound scratch assay analysis of the impact of ITGA3 knockdown on cell migration (***P < 0.001, *P < 0.05, compared with the respective control). **(B)** Transwell assay analysis of the impact of ITGA3 knockdown on cell invasion of PTC cell lines.

Tumor cell invasion and migration were the important features that associated with tumor progression. To investigate whether ITGA3 affects the migration and invasion of PTC cells, we performed scratch assays and transwell assays. As shown in **Figures 11A, B**, knockdown of ITGA3 significantly reduced the migration and invasion capability BCPAP cells. These results suggested that ITGA3 enhances the capacity of migration and invasion of PTC cells.

## Discussion

PTC is the most common pathological type of thyroid cancer. Lymph node metastasis is an important factor affecting the prognosis. How to reduce the recurrence of thyroid cancer after surgery is still a topic worth discussing currently. Surgical treatment is usually the first choice for PTC. The primary tumor size and the lymph node metastasis status were the determinant factors for the extent of surgery (22). Therefore, knowing lymph node metastasis status before surgery is very important for guiding surgical treatment. If a molecular marker could effectively predict the status of lymph node metastasis in PTC, it will be beneficial for the clinical treatment.

ITGs are heterodimeric receptors expressed on the cell membrane and consist of α and β subunits. There are many types of ITGs. At least 18 kinds of α subunits and 8 kinds of β subunits have been identified. Their function is to mediate ECM and immunoglobulin superfamily molecule adhesion (11, 23). ITG preferentially binds to its matching ECM protein, and the ITG expressed in the cell determines the degree of adhesion and migration of the cell on different substrates. During the connection with ECM, ITG gathers on the membrane and recruits various signaling proteins and adaptor proteins to form focal adhesions, which can activate focal adhesion kinases (FAKs) and Src family kinases (SFKs) (11). The expression of ITG is very different between normal tissues and tumor tissues. Various ITGs play a role in promoting tumor progression, including αvβ3, α2β3, and α2β1 (23). Since many solid tumors originate from epithelial cells, the ITG expressed by epithelial cells is usually retained in the tumor. These ITGs usually mediate the adhesion of epithelial cells to the basement membrane but may contribute to the migration, proliferation, and survival of tumor cells (11). The ITGA3 gene encodes the ITG α-3 subunit. ITGA3 interacts with members of the ECM protein and laminin family, and their high expression in tumors is related to cell proliferation, invasion, and metastasis (24, 25). Although the clinical value of this gene in thyroid cancer is undetermined, recent studies have revealed that ITGA3 plays an important role in multiple cancers; it is highly expressed in bladder cancer, pancreatic cancer, and endometrial cancer and was correlated with poor prognosis (13, 26–28).

In our study, we examined the differentially expressed gene ITGA3 of PTC using the Oncomine gene database. The expression of ITGA3 in PTC was significantly greater than that in normal tissue, except the follicular subtype, of which the encapsulated noninvasive form was considered as the least invasive (29). To explore the correlation between ITGA3 gene expression and the clinicopathologic characteristics in thyroid cancer, dataset from TCGA was used for further analysis. We found that tall cell variant PTC expressed the highest level of ITGA3, followed by classical PTC, and the follicular variant subtype expressed the lowest. In the cohort of PTC from TCGA dataset, the expression level of ITGA3 in patients with lymph node metastasis was significantly higher than that without lymph node metastasis, presenting a positive correlation of ITGA3 gene expression with PTC lymph node metastasis. Multivariate analysis using logistic regression model showed that high expression of ITGA3 and advanced pathologic T were the independent risk factors that promote the recurrence of PTC. The ROC analysis was conducted to evaluate the expression of ITGA3 for predicting lymph node metastasis, local invasion, and tumor recurrence. The results suggested that high expression of ITGA3 had a moderate diagnostic significance for lymph node metastasis of central neck and low diagnostic significance for regional invasion and recurrence.

Furthermore, we surveyed the prognostic value of ITGA3 using survival analysis. The Kaplan–Meier model was used for RFS analysis. It turned out that high expression of ITGA3 was connected with poor RFS. Besides, our analysis found that histological type, tumor regional invasion, and lymph node metastasis were the risk factors that influence RFS of PTC patients. Therefore, we speculated that high expression of ITGA3 gene could be used as a predictor for lymph node metastasis and recurrence of PTC. The clinical application of ITGA3 gene test should be encouraged. Cervical lymph node dissection should be performed in patients with high levels of ITGA3 gene expression.

The co-expressed genes with ITGA3 were analyzed using GEPIA database to explore ITGA3-related altered pathways in thyroid carcinoma. The co-expressed profile was confirmed with heat map plotted by UCSC Xena web-based tools. Analysis based on TCGA database suggested that the expression of ERBB3, a member of epidermal growth factor receptor (EGFR) family, was highly co-altered with ITGA3 expression among the positively correlated genes. Amplification of ERBB3 had been reported in numerous cancers, including ovarian cancer, gastric cancer, breast cancer, and melanoma, and was correlated with cancer progression and poor prognosis (30–33). In tumor cells, cooperation between ITGs and members of the EGFR family was able to promote tumor initiation, proliferation, migration, and invasion (34–36). Moreover, pathway enrichment analysis using GSEA revealed that several cancer-related pathways were involved in ITGA3 high expression group, including MAPK pathway and ERBB pathway. The MAPK signaling is one of the most commonly dysregulated pathways found in multiple cancers (37, 38). The activation of MAPK pathway by RAF proteins leads to abnormal differentiation, proliferation and apoptosis, and cancer development (39, 40). As a result, ITGA3 should be taken seriously that it may act as a potential therapeutic target in radioactive iodine-refractory PTC.

We examined the expression of ITGA3 in PTC and normal thyroid tissues by immunohistochemical analysis. We found that ITGA3 was highly expressed in PTC tissues compared to adjacent normal gland, and ITGA3 expression was higher in recurrent PTC than in no-recurrent PTC, which was consistent with the bioinformatics analysis results. In our cell experiments, knockdown of ITGA3 suppressed the proliferation, invasion, and migration capacity of thyroid cancer cells, suggesting ITGA3 may play an active role in PTC invasion and metastasis.

In conclusion, our bioinformatics analysis and experiments demonstrate that ITGA3 is highly expressed in papillary thyroid carcinoma and is related to the progression of thyroid cancer and poor prognosis. ITGA3 is an independent risk factor of papillary thyroid cancer recurrence. The molecular mechanism of ITGA3 involved in the progression of thyroid cancer needs to be clarified by further experiments.

## Data Availability Statement

The original contributions presented in the study are included in the article/supplementary material. Further inquiries can be directed to the corresponding author.

## Ethics Statement

The studies involving human participants were reviewed and approved by the Ethics Committee of the Affiliated Hospital of Putian University. The ethics committee waived the requirement of written informed consent for participation.

## Author Contributions

GZ contributed to the article drafting and data acquisition and analysis. BL contributed to research design and making important suggestions. YL contributed to study supervision and article revising. All authors have given final approval of the version to be published.

## Funding

The work was supported by the Research project of Putian University (Grant No. 2017049) and science and technology planning project of Putian (Grant No. 2020S3F003).

## Conflict of Interest

The authors declare that the research was conducted in the absence of any commercial or financial relationships that could be construed as a potential conflict of interest.

## Publisher’s Note

All claims expressed in this article are solely those of the authors and do not necessarily represent those of their affiliated organizations, or those of the publisher, the editors and the reviewers. Any product that may be evaluated in this article, or claim that may be made by its manufacturer, is not guaranteed or endorsed by the publisher.
